# Editorial: Metabolomics in the Study of Unconventional Biological Matrices

**DOI:** 10.3389/fchem.2021.736661

**Published:** 2021-08-03

**Authors:** Elena Sánchez-López, Tommaso Lomonaco, Masahiro Sugimoto, Yunping Qiu, Beatrice Campanella

**Affiliations:** ^1^ Department of Human Genetics, Leiden University Medical Center, Leiden, Netherlands; ^2^ Department of Chemistry and Industrial Chemistry, University of Pisa, Pisa, Italy; ^3^ Research and Development Center for Minimally Invasive Therapies, Tokyo Medical University, Shinjuku, Japan; ^4^ Department of Medicine, Albert Einstein College of Medicine, Bronx, NY, United States; ^5^ Institute of Chemistry of Organometallic Compounds, National Research Council, Pisa, Italy

**Keywords:** metabolomics, unconventional matrices, biomarkers, mass spectrometry, sample preparation

Metabolomics focuses on the study of small molecules, i.e. metabolites, produced by living organisms, providing rich information on their state under specific conditions at specific points in time. Well-established matrices such as urine, serum, and plasma are widely used in clinical and environmental studies aiming to discover new biomarkers linked to several pathologies or to identify the alteration of metabolic cycles due to the effect of external agents. Nowadays, unconventional biological matrices have attracted increasing attention in the field of metabolomics as a means to unravel new molecular mechanisms. Moreover, since metabolites represent the final response to environmental stress, matrices such as animal and vegetable-derived material, whose metabolome remains largely unexplored, could also be exploited to assess the effects of concerning contaminants on an organism.

These unconventional matrices bring challenging issues such as the absence of standardized collection protocols, the absolute quantity of collected material, as well as the lack of standardized reference materials and normalization methods for the quantified analytes. Improvements in data integration and data analysis workflows are also crucial to provide a clear understanding of the mechanisms and pathways involved in healthy and diseased physiological processes, as well as the combination of metabolomics and software dedicated to identifying cellular pathways.

In this Research Topic, we present a collection of five original research papers showing recent advances both in the untargeted and targeted metabolomics field related to the study of unconventional samples. Sweat is an innovative and unconventional matrix used to monitor health status. Sweat composition is influenced by several factors that may limit its application in the clinical setting. Here, Harshmann et al. performed a proof-of-concept study aimed at evaluating the impact of nutritional supplementation on the sweat metabolome. They collected sweat samples from the forearms of 13 participants in a 12-weeks exercise program that foresees the ingestion of low or high nutritional supplementation twice daily. The authors demonstrated that ingestion of regular nutritional supplementation semi-quantitatively impacts the sweat metabolome. Moreover, they highlighted the use of dried powder mass as a convenient strategy for metabolomic data normalization from sweat samples. Another interesting unconventional matrix is hair. Hair is an exceptional biological sample that can adsorb different exposure such as environmental contaminants and therefore is ideal to study the exposome. Chen et al. conducted an untargeted metabolomics study of human hair samples using LC-MS. They compared the profiles at the samples before and after a 2-days exposure exercise and found the significant differences of the part of the profiles, which suggests the need for standardization in sample collection to eliminate the unexpected bias.

*In vitro* studies are considered a great alternative to animal and human studies since they can help to unravel biochemical mechanisms without the ethical requirements of the *in vivo* studies. In their work, Campanella et al. analyzed, for the first time, the extracellular metabolism of living immortalized hippocampal HN9.10e cells, a murine *in vitro* model of one of the most vulnerable regions within the central nervous system. They characterized the normal basal status of this cell line, paying particular interest to the central carbon metabolism using a targeted metabolomics approach. It is clear that to get a robust biological interpretation, sample preparation is a paramount step to take into account in any metabolomics study since it has a vast impact on the metabolic composition. In this regard, Erngren et al. evaluated the effect of different sampling conditions on the metabolic profile of the marine sponge *Geodia barretti* using both HILIC and RPLC, in positive and negative ionization modes. Using this wide approach in an untargeted fashion, authors found that flash-frozen with liquid nitrogen was the sampling strategy that provided the highest metabolite intensity also with low variability among replicates. The results found in this study will serve to help the sponge metabolomics community in establishing guidelines for a practical consensus.

Metabolomics studies generate a large size of data that needs sophisticated tools to illustrate the biological information behind it. Zhou et al. developed an R-based free web-based integrated Metabolomics Analysis Platform (iMAP) for metabolomics data, an advanced version of their desktop platform IP4M (integrated Platform for Metabolomics Data Analysis). In this new platform, the authors integrated the most useful features for metabolomics data including feature selection, training and validation for predictive models, network construction, and visualization. As a proof-of-principle authors applied this tool to two independent datasets, one of them including clinical stool samples.

In summary, this collection includes recent contributions in the metabolomics field, with a special focus on advancing the characterization of living organisms using unconventional biological samples. Particularly advances in sample preparation, sample analysis, and data analysis have been of special interest.

